# Supplementary material for: A phosphatidylinositol 4,5-bisphosphate redistribution-based sensing mechanism initiates a phagocytosis programing
Source: Nat Commun. 2018 Oct 15;9:4259. doi: 10.1038/s41467-018-06744-7 (PMC6189171; doi:10.1038/s41467-018-06744-7)
Supplement: Supplementary file 1 — Supplementary Information [file 41467_2018_6744_MOESM1_ESM.pdf]

Supplementary Information

A phosphatidylinositol 4,5-bisphosphate redistribution-based sensing mechanism initiates a phagocytosis programing

Mu, Tu, *et al*.

Supplementary Methods

Real-time PCR

Real-time PCR (or quantitative PCR) was performed according to the instructions of TransStart® Green qPCR SuperMix (AQ101, Transgen Biotech). Primers for real-time PCR were as follows: Msn: sense 5'-TCCCAGTTGGAAATGGCTCG-3'; antisense 5'-GCTCTGCCACATGAGGTGTA-3'; Ezr: sense 5'-ACAGCAGTTGGAAACCG-3'; antisense 5'-GGCCTCCAGACGTTCAG-3'; Rdx: sense 5'-CTCTTACCACAGCGTGTTTTGG-3'; antisense 5'-TTCCCTTAGCATCCCTCTGTGT-3'; Merlin: sense 5'-CATACCGAGCTTCGACATTAT-3'; antisense 5'-CTTGCTCTTCTCCATGTACTC-3'; Frmd4a: sense 5'-CAACGAGAACCGCATCAA-3'; antisense 5'-GTCTTCGCTGGCGATATTT-3'; Frmd4b: sense 5'-GGGACCACGTTCAAGTTAG-3'; antisense 5'-CAACCAGCTTCTGCTGTAT-3'; Csf1r: sense 5'-CATGGCCTTCCTTGCTTCTAA-3'; antisense 5'-TGCCGTAGGACCACACATCA-3'; Fcer1g: sense 5'-ATATCCTGGATGCTGTCCTG-3'; antisense 5'-TCTCATATGTCTCCTGGCTC-3'; Hmox1: sense 5'-GAATCGAGCAGAACCAGCCT-3'; antisense 5'-CTCAGCATTCTCGGCTTGGA-3'; Lcp1: sense 5'-ACTGAGAATTCAAGTCTGTCACCT-3'; antisense 5'-AGCTGATGTATCCGTTGCCA-3'; Ndrg1: sense 5'-CCTCAACGACATGAACCCGA-3'; antisense 5'-TGCAAAGTGACAGTGTGGGT-3'; Tyrobp: sense 5'-GAAGGGACCCGGAAACAACA-3'; antisense 5'-CTGATGGGCATAGAGTGGGC-3'.

Atomic force microscopy-based single cell force spectroscopy

Experiments were performed as previously described using a JPK CellHesion unit [1]. In brief, a clean cantilever was placed on top of a glass block holder. Using a video camera attached to AFM, a laser was focused on the cantilever and properly aligned with respect to the position detector. Suitable

silica crystals or polystyrene beads were placed directly onto the surface of a glass coverslip (diameter 2.4 cm), equal parts of the AB epoxy were mixed together and spread thinly at the side of the same glass coverslip. The clean cantilever gradually was moved to approach the glue so that only the very edge of the tip was over the glue. It was then lowered until a change in vertical deflection was observed and the tip had visible glue drawn up. Then the cantilever was pulled away from the glue until an observable snapping motion of the tip was observed, and was moved to individual silica crystals or polystyrene beads. The cantilever with glue was slowly lowered until the tip was in focus, and another deflection of the laser would be observed once the tip was in contact with the target. When raising the cantilever, the silica crystal or polystyrene bead on the glass coverslip should disappear if there was a successful attachment. To be certain that the target was glued to the tip, it was also examined under a compound light microscope. Functionalized cantilever tips were left to harden in 37°C for 30 mins or room temperature over night for further curing.

All instruments of AFM were turned on 30 mins before single cell force spectroscopy (SCFS) experiments to ensure thermal equilibration and reduce drift during experiments. The coverslip seeded with DC 2.4 cells was mounted onto a customized holder for AFM. The laser was focused on the back of the cantilever and aligned according to the guidelines of the AFM manufacturer. To calibrate the cantilever, a force-distance curve was recorded. Cantilever sensitivity and spring constant were determined using the routines incorporated in the AFM control software. For force reading, the cantilever was retracted approximately 50 μm from the surface. The apex of cantilever was manually positioned directly above a suitable cell. All measurements were performed with the JPK CellHesion 200 in relative force feedback contact mode (constant force 1.5 nN, contact time 5 s, pulling length 50 um, and constant speed 5 μm/s). Force curves were normalized and analyzed using JPK data processing software. Maximum binding forces were calculated and plotted over time.

Generalized Polarization analysis

For GP analysis, cells were excited at 405 nm, and images were recorded with 450nm and 525 nm for ordered and disordered membranes, respectively. GP images were calculated via radiometric imaging methods detailed in Owens et al. [2]. Specifically, GP= (I450-G×I525)/(I450-G×I525), where the sensitivity correction factor G was experimentally determined using a solution 10 μM c-Laurdan in DMSO following a procedure described previously [3].

Supplementary Figures

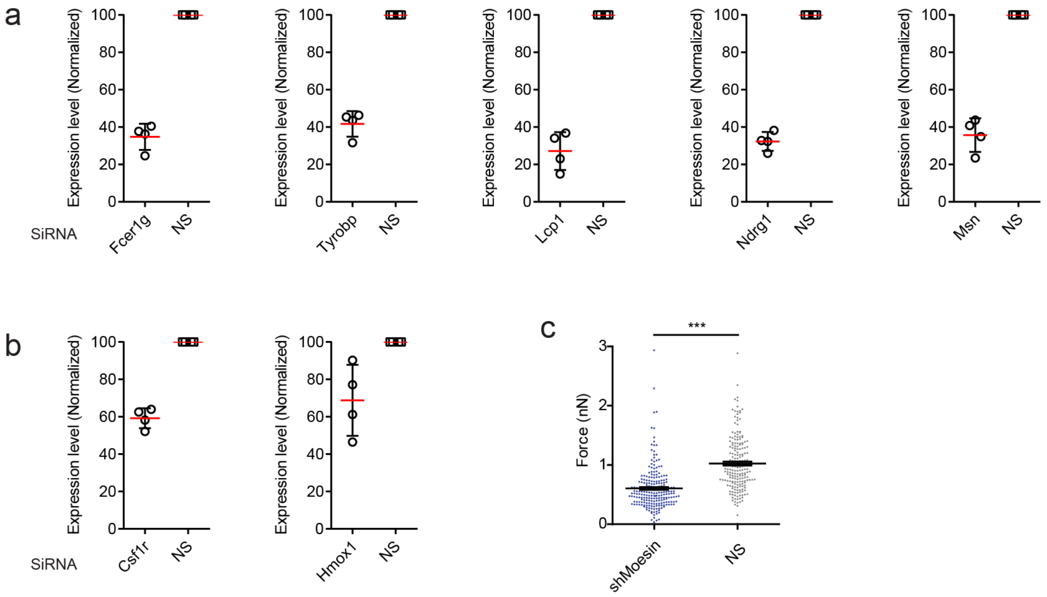

Supplementary Figure 1. Screening for ITAM-containing molecules. (**a**) QPCR of DC2.4 cells transfected with indicated siRNA, or Non-specific siRNA (NS, no homologous matching in mouse genome, used henceforth in subsequent figures) as control. The ratios of mRNA levels over GAPDH with indicated siRNA or control siRNA transfection are shown. Data were presented as mean±s.d. N=4. (**b**) QPCR of DC2.4 cells transfected with Csf1r, Hmox1 siRNA or NS siRNA control. The ratios of mRNA levels over GAPDH with indicated siRNA or control siRNA transfection are shown. Data are presented as mean±s.d. N=4. (**c**) AFM force measurement of Moesin-KD or control DC2.4 in contact with silica crystals. n=190, N=3. ***$p<0.001$ by Student's *t* test.

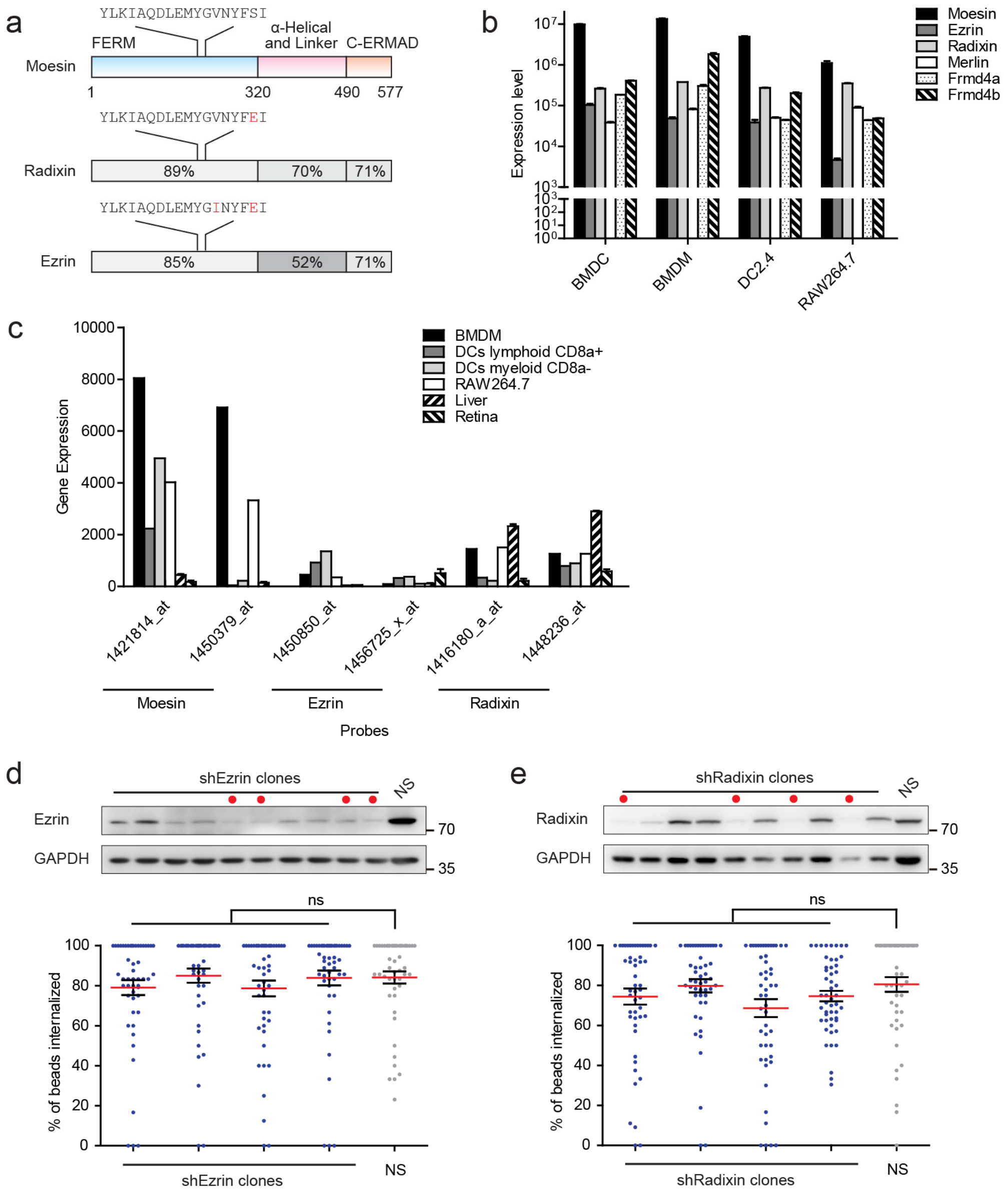

Supplementary Figure 2. Radixin and Ezrin are not involved in receptor-independent phagocytosis. (**a**) Molecular structure of Moesin, Radixin and Ezrin. Domains and sequences of ITAM motif are shown. Amino acids in ITAM different from Moesin are labeled in red. (**b**) Absolute quantification PCR of ERM family member mRNA level derived from indicated cells. n=3, N=3. (**c**) Relative expression levels of three ERM proteins, revealed by the indicated probes from BioGPS (http://biogps.org) database, in mouse phagocytes and tissues. (**d**) Ezrin and (**e**) Radixin shRNA-transfected DC2.4 cell single clones or NS control were immunoblotted with antibodies against corresponding proteins, and GAPDH (upper). The phagocytosis efficiency of the clones with high KD efficiency (indicated by red dot in upper panels, and used in the assay in the lower panels) is shown comparing to NS control (lower). n=50, N=3. ns p>0.5 by one-way ANOVA with Scheffé post-hoc.

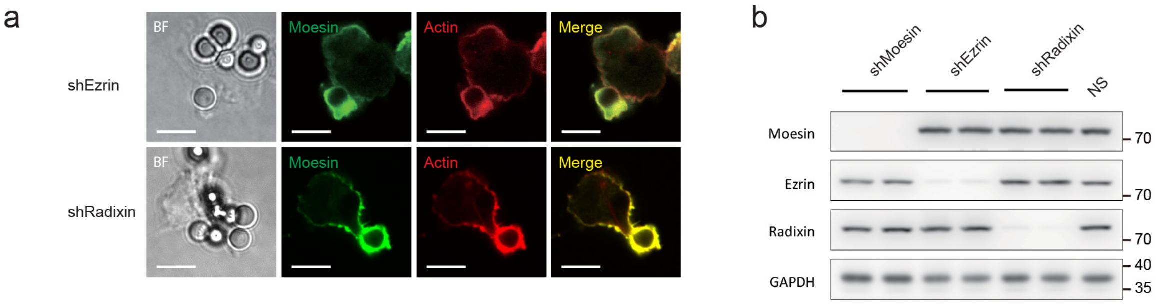

Supplementary Figure 3. Anti-Moesin antibodies are specific. (**a**) Moesin (green) was visualized with anti-Moesin antibodies followed by secondary antibodies and actin cytoskeleton (red) was visualized by Alexa568-Phalloidin on Ezrin (upper) or Radixin (lower) shRNA knockdown DC2.4 cells incubated with 3 µm polystyrene beads. Confocal microscopy was performed to obtain the fluorescence images. Scale bars, 5 µm. (**b**) WB analysis of protein levels of the ERM members in shRNA knockdown single clones, compared with NS control cells.

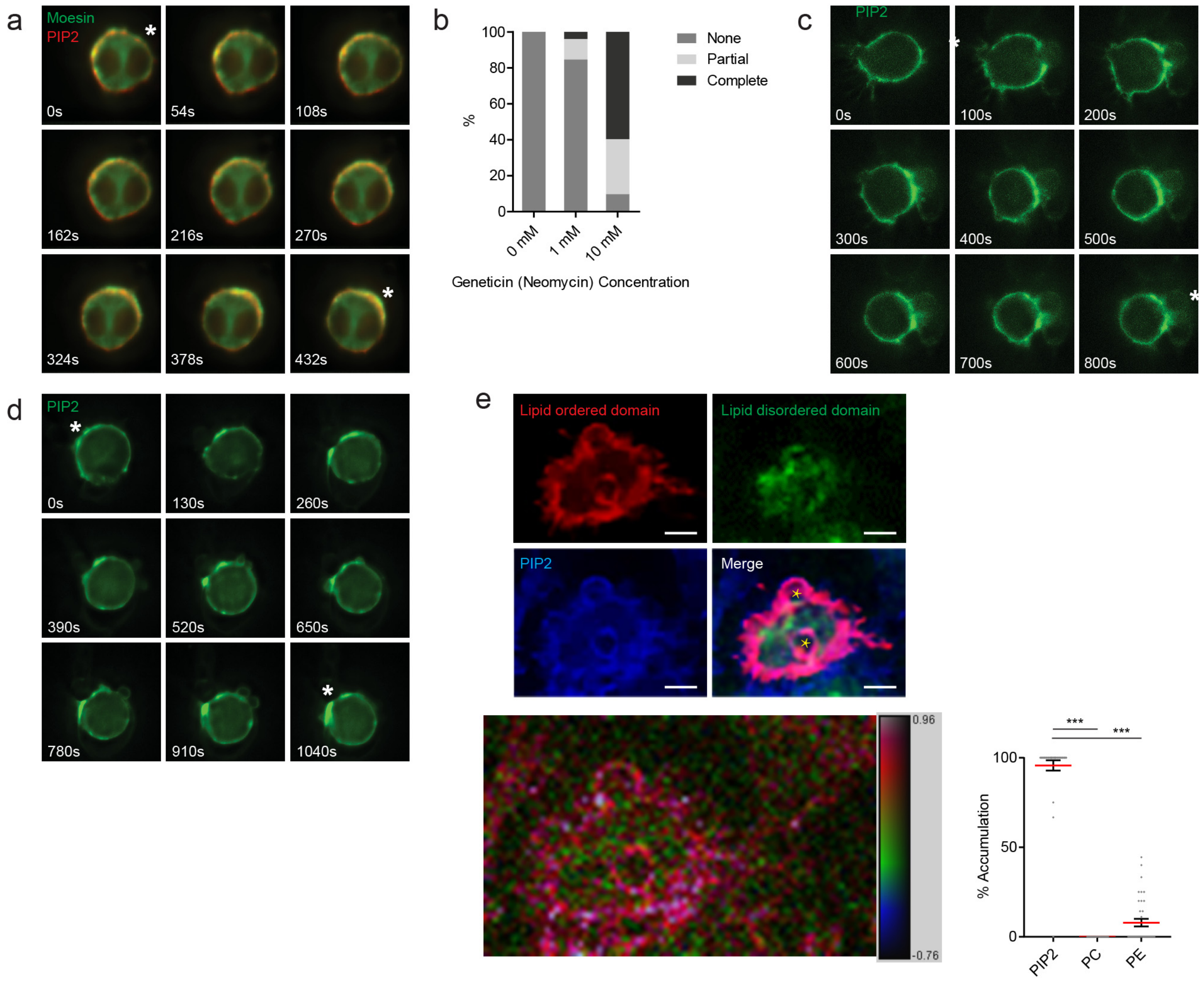

Supplementary Figure 4. Moesin signaling is downstream of PIP2 sorting driven by solid structures. (**a**) Fluorescence images of Moesin-EGFP and PH-PLCδ-mCherry-expressing RAW264.7 cells in contact with a naked polystyrene bead (indicated with "*") glued to an AFM cantilever at indicated time points. (**b**) Efficiency of PIP2 sequestration was determined by treating stable PH-PLCδ-GFP-expressing RAW264.7 cells with 0, 1 or 10 mM Geneticin and counting the different types of PH-PLCδ-GFP subcellular distribution. Cells were categorized into three different phenotypes. None: no visible sequestration of PIP2, Partial: PIP2 shows increased presence in the cytosol while with significant retention in the plasma membrane. Complete: no visible retention in the membrane. (**c**) and (**d**) Fluorescence images of PH-PLC -GFP-expressing RAW264.7 and HEK293T cells, respectively, in contact with a naked polystyrene bead (indicated with "*") glued to an AFM cantilever at indicated time points. (**e**) Left: PH-PLCδ-mCherry-expressing RAW264.7 cells were cultured on PDMS micropatterns (indicated with yellow '*') are incubated with one μM c-Laurdan. TopFluor-TMR PC and PE were used as controls for other lipid species. Middle: For GP analysis, cells were excited at 405 nm, and images were recorded with 450nm and 525 nm for ordered (blue) and disordered (green) membrane domains, respectively. mCherry signals were also collected at the same time to assess PIP2 sorting (red). GP images were calculated per Owens et al.[2]. Scale bars, 5μm. Right: The number of patterns with high membrane orders (GP>0.5) and lipid accumulation were counted for every individual cell. Comparisons were made between different lipid species (right). n⩾38, N=3. ***p<0.001 by one-way ANOVA with Scheffé post-hoc.

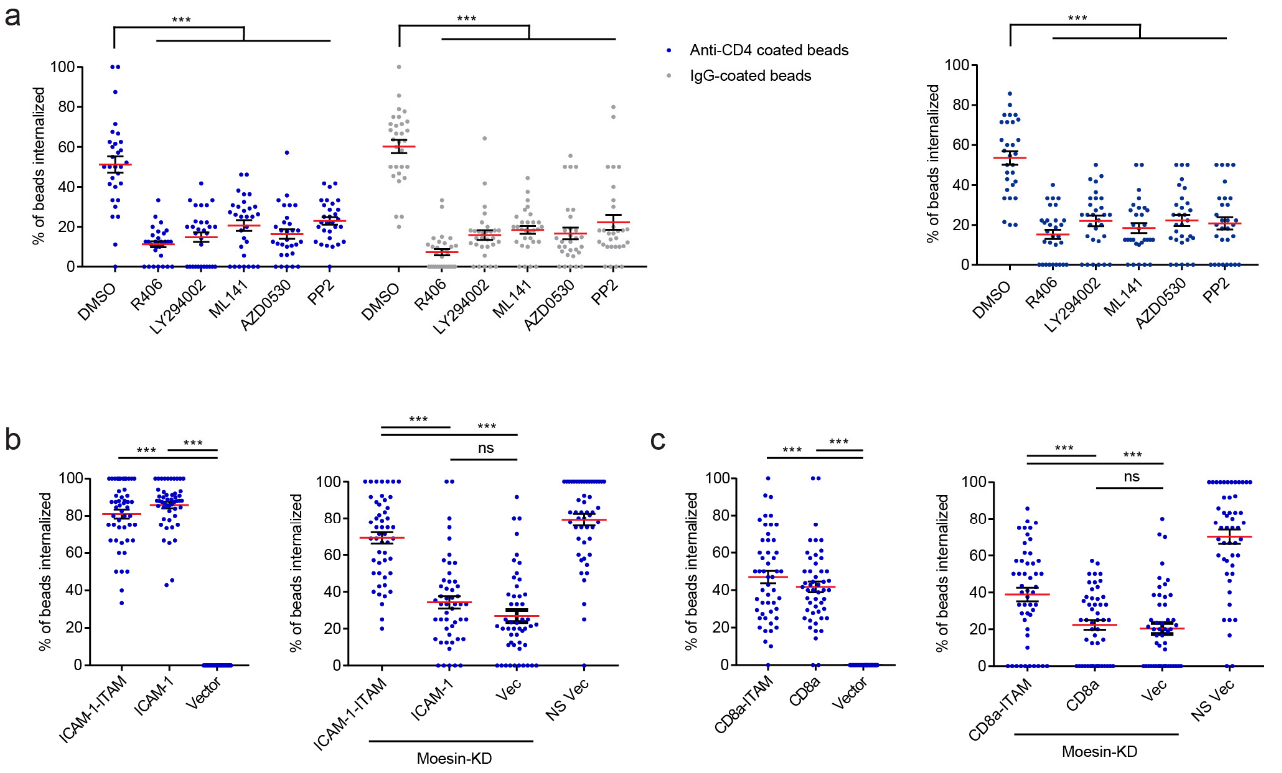

Supplementary Figure 5. Common features shared by different pathways in solid structure phagocytosis. (**a**) Left: Phagocytosis assays were performed on CD4-transfected Cos-1 cells with IgG-coated or anti-CD4 coated 3μm beads in the presence of specific Syk, PI3K and Cdc42 inhibitors R406 (10 μM), LY294002 (50 μM), ML141 (10 μM), as well as two Src inhibitors AZD0530 (10 μM) and PP2 (10 μM). Right: The same assay was performed on CD4-ITAM-transfected Moesin-KD DC2.4 cells. n=50, N=4. ***p<0.001by one-way ANOVA with Scheffé post-hoc. (**b**) Left: Cos-1 cells were transfected with ICAM-1, ICAM-1-ITAM chimeric molecule or empty vector control by transient overexpression, and the same phagocytosis assay as in a was performed using anti-ICAM-1 coated beads. Right: identical to the left, except that Moesin-KD DC2.4 cells were rescued with the constructs. Non-specific shRNA (NS) transfected cells were also used as the control for Moesin KD. ***p<0.001and ns p>0.05 by one-way ANOVA with Scheffé post-hoc. (**c**) Both panels are identical to **b** except that CD8α construct and antibody were used in place of ICAM-1 counterparts. ***p<0.001and ns p>0.05 by one-way ANOVA with Scheffé post-hoc.

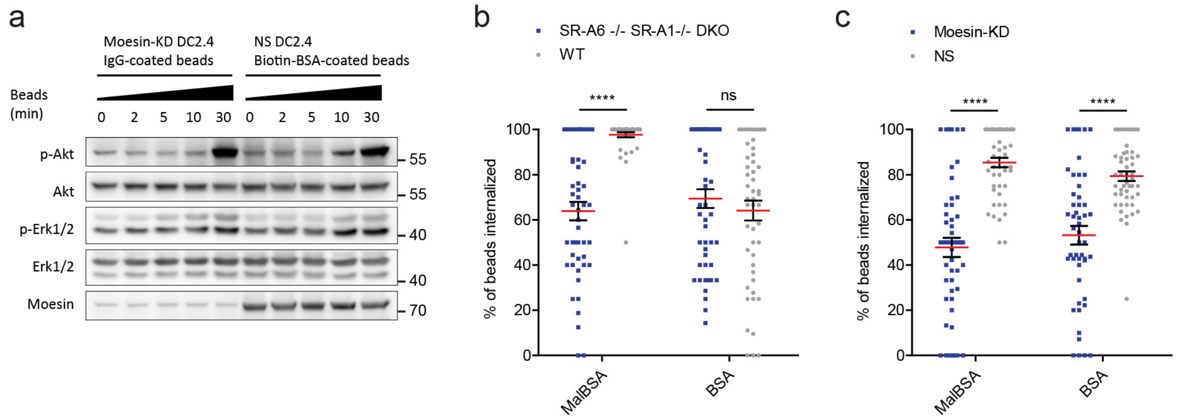

Supplementary Figure 6. Impact of PIP2-Moesin-Syk pathway in other solid structure phagocytosis. (**a**) Moesin-KD and control DC2.4 cells were treated with IgG-labeled or Biotin-labeled beads for indicated times. Total cell lysates were subjected to immunoblotting with the indicated antibodies. (**b**) Phagocytosis assay was performed on C57BL/6 WT and SR-A6 $^{-/-}$SR-AI $^{-/-}$ BMDM with MalBSA, a SR-A6 ligand, and BSA-coated beads. n=50, N=3. (**c**) Phagocytosis assay was performed on NS and shMoesin DC2.4 cells with MalBSA, and BSA-coated beads. n=50, N=3. ****p<0.0001 and ns p>0.05 by one-way ANOVA with Scheffé post-hoc.

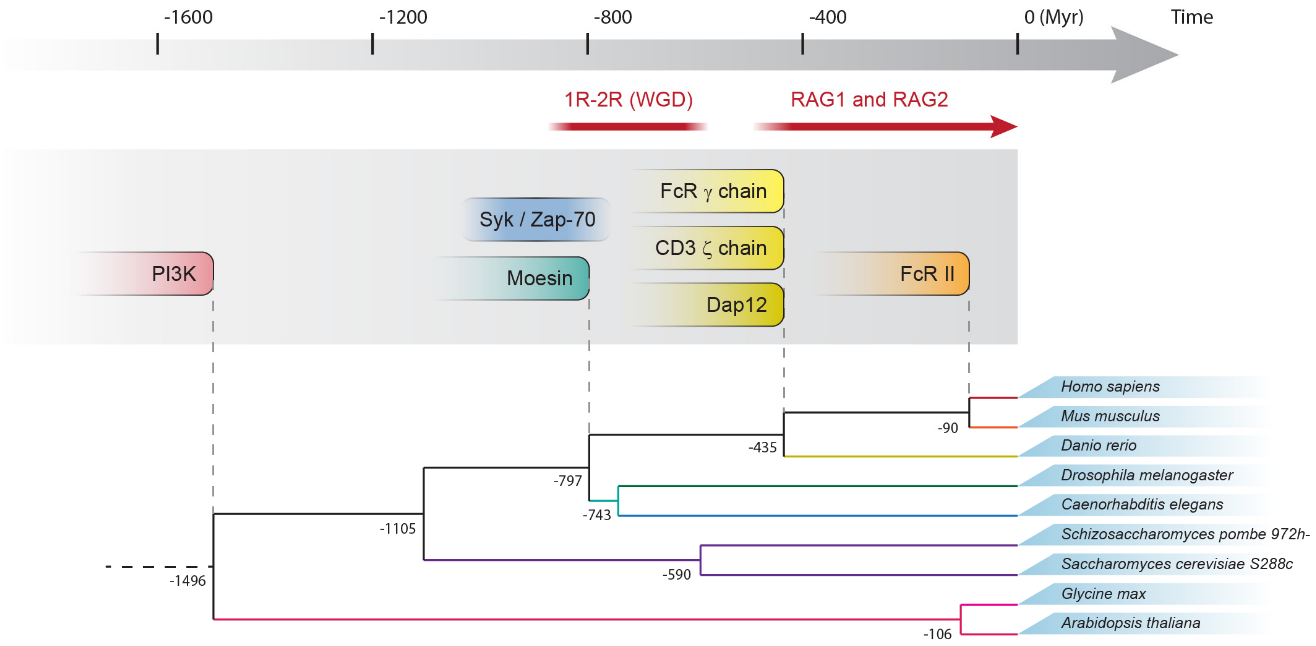

Supplementary Figure 7. A simplified overview of the latest time of hypothetic origin of indicatedproteins, derivative of Fig. 7.

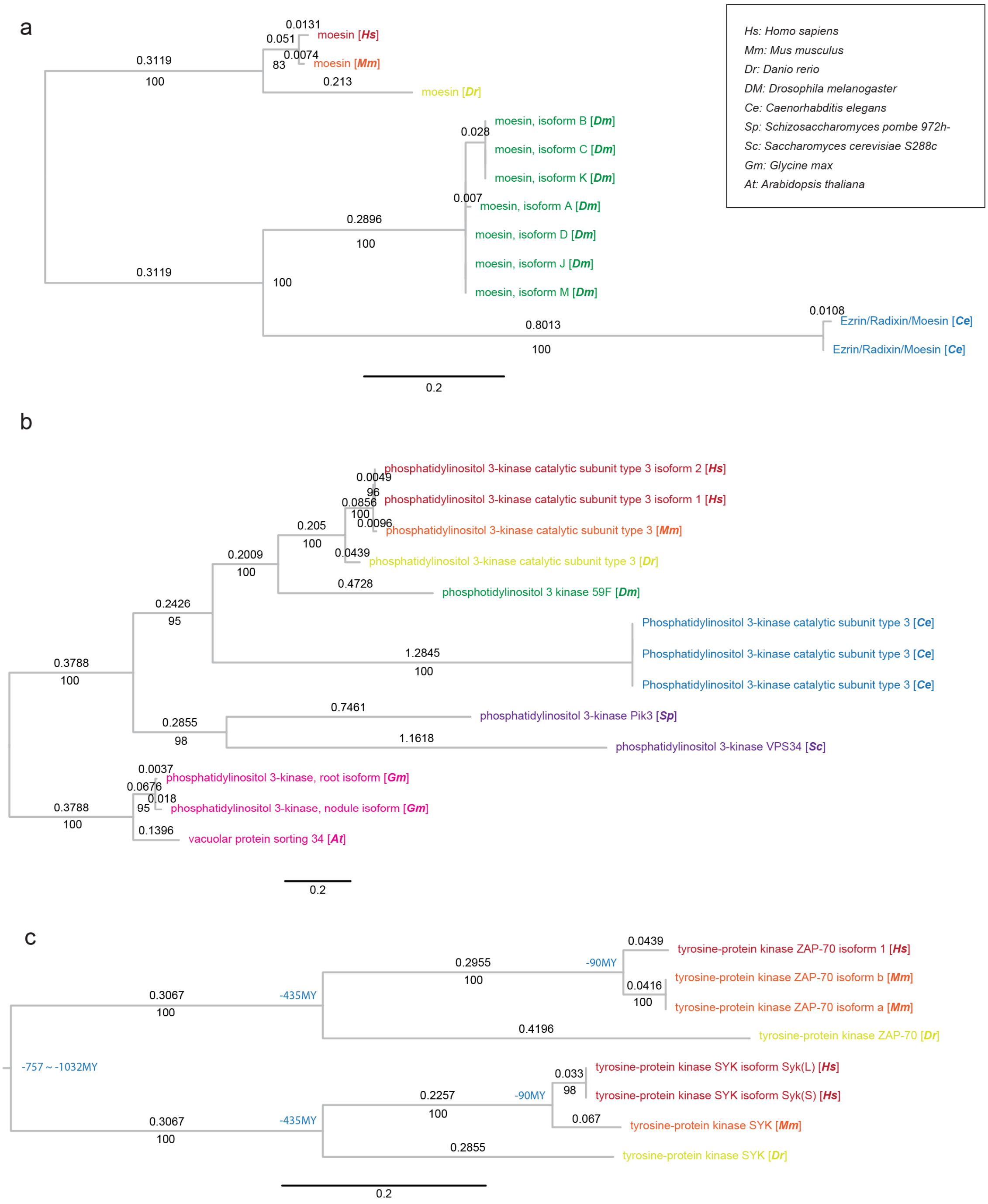

Supplementary Figure 8. Phylogenetic trees of key phagocytic signaling proteins. These trees include (**a**) ERM, (**b**) PI3K catalytic subunit and (**c**) Syk/ZAP-70. Numbers above branches show branch lengths in the unit of number of substitution per site. Numbers below branches represent bootstrap support in percentage from 100 replicates, and values less than 50 are not shown. The scale bar is in the unit of substitutions per site. The data were gathered to produce **Figure 7**.

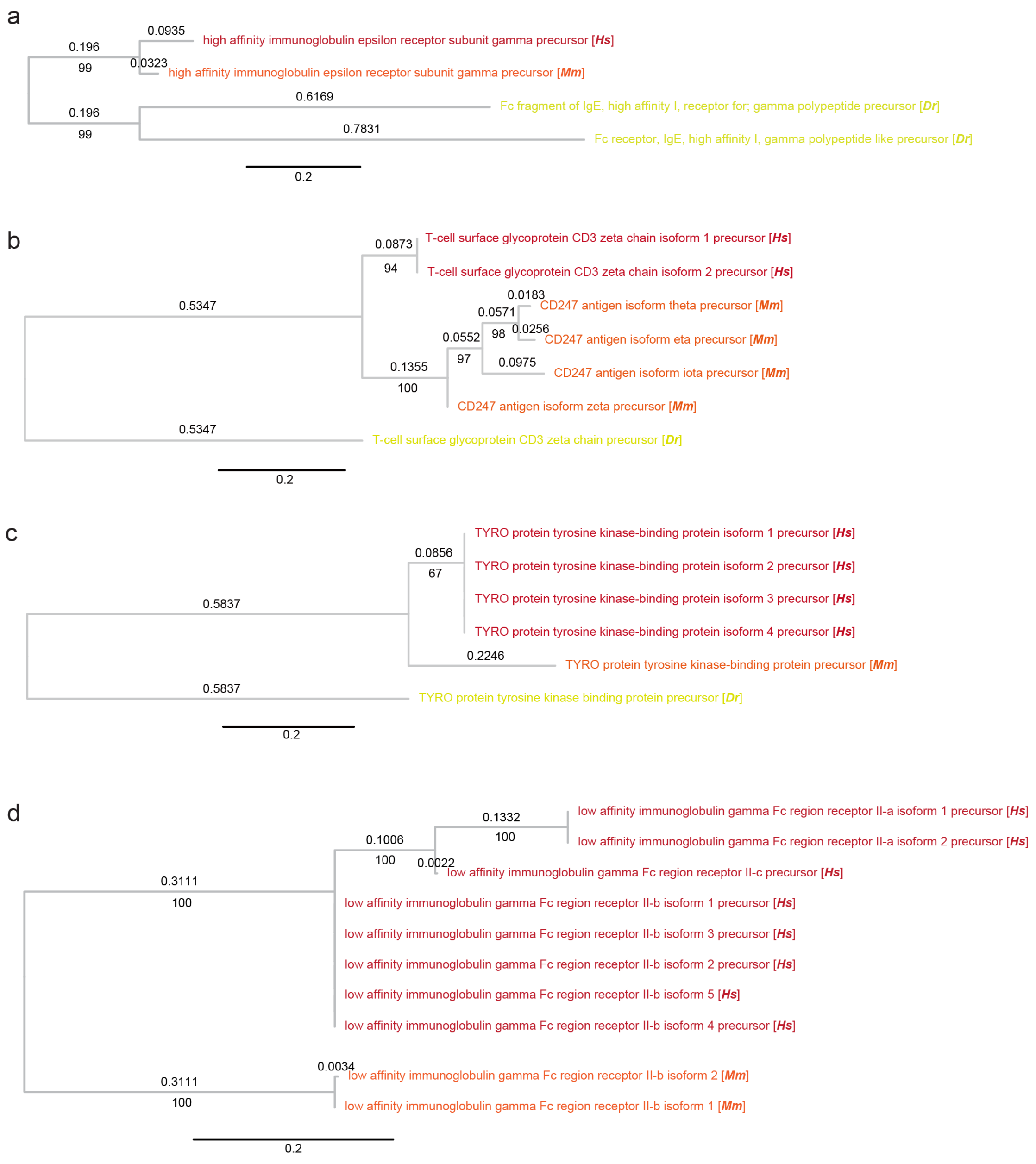

Supplementary Figure 9. Phylogenetic trees of key immune signaling proteins. These trees include (**a**) FcR common γ chain, (**b**) CD3 ζ chain, (**c**) DAP12 and (**d**) FcRs. Numbers above branches showbranch lengths in the unit of number of substitution per site. Numbers below branches representbootstrap support in percentage from 100 replicates, and values less than 50 are not shown. The scalebar is in the unit of substitutions per site. The data were gathered to produce **Figure 7**.

a

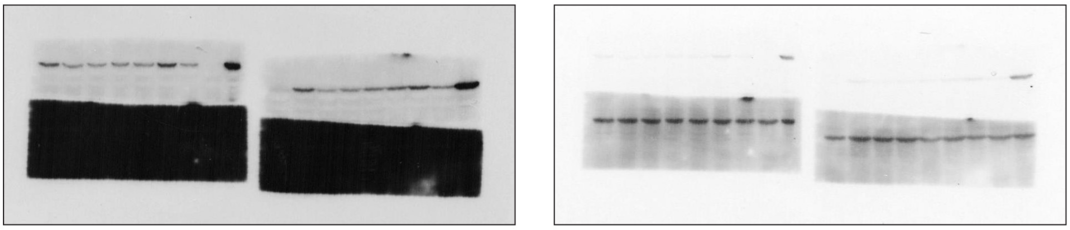

b

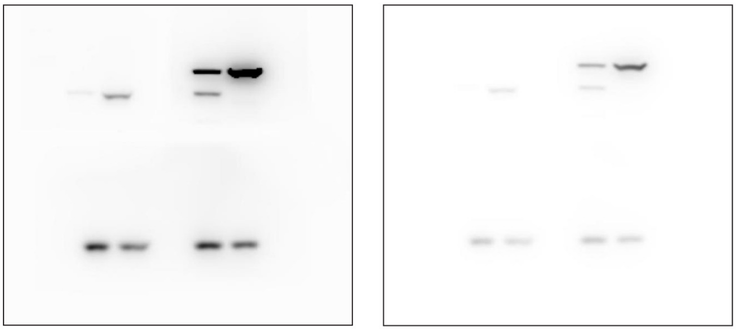

c

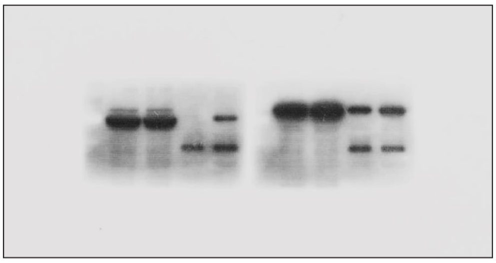

d

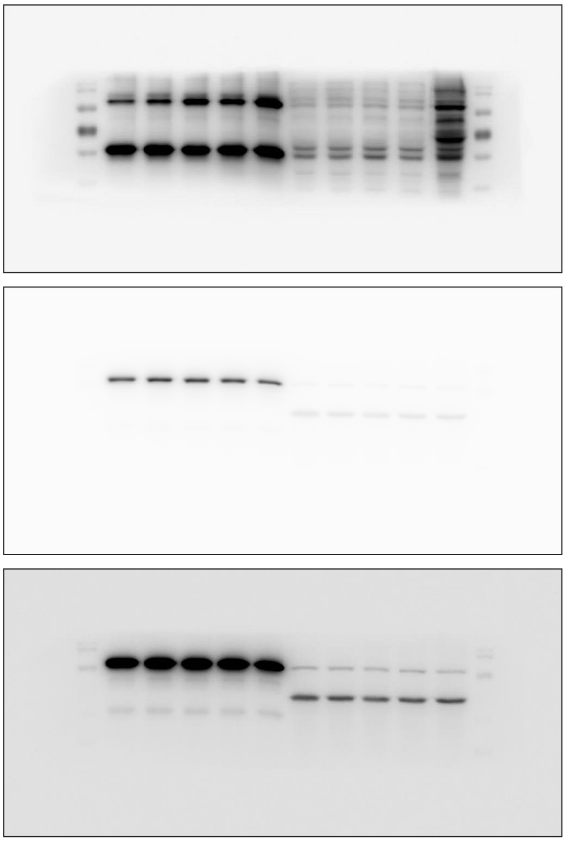

Supplementary Figure 10. Original immunoblots of Figure 1b, 2a, b and c. (a), Uncropped Figure 1b. (b), Uncropped Figure 2a. (c), Uncropped Figure 2b. (d), Uncropped Figure 2c.

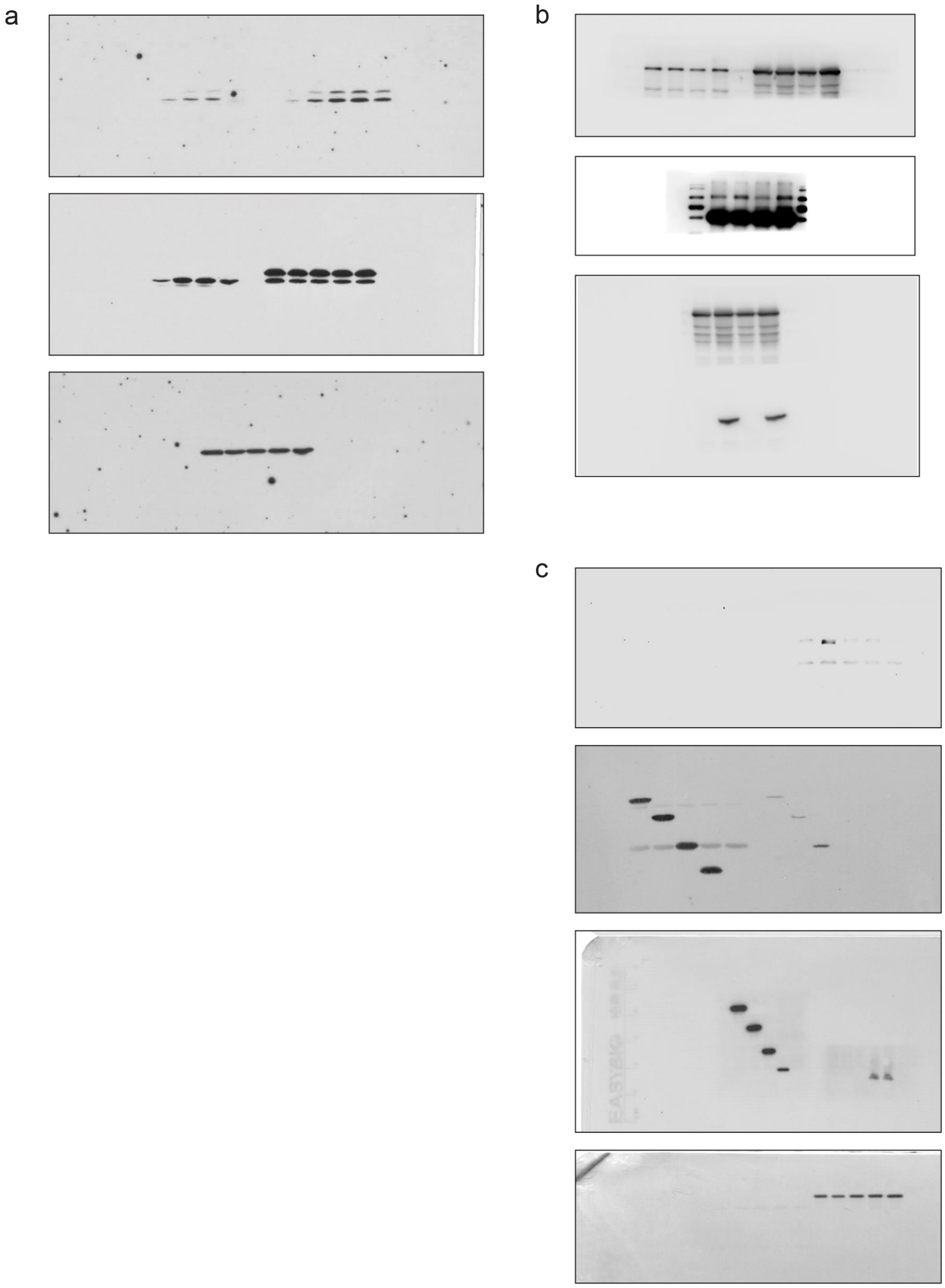

Supplementary Figure 11. Original immunoblots of Figure 2d, e , g and Figure 7c. (a), Uncropped Figure 2d. (b), Uncropped Figure 2e. (c), Uncropped Figure 2g.

Supplementary References

1 Flach, T. L. *et al.* Alum interaction with dendritic cell membrane lipids is essential for its adjuvanticity. *Nature medicine* **17**, 479-U121, doi:10.1038/nm.2306 (2011).

2 Owen, D. M., Rentero, C., Magenau, A., Abu-Siniyeh, A. & Gaus, K. Quantitative imaging of membrane lipid order in cells and organisms. *Nat Protoc* **7**, 24-35, doi:10.1038/nprot.2011.419 (2011).

3 Kim, H. M. *et al.* A two-photon fluorescent probe for lipid raft imaging: C-laurdan. *Chembiochem : a European journal of chemical biology* **8**, 553-559, doi:10.1002/cbic.200700003 (2007).
