# Supplementary material for: A phosphatidylinositol 4,5-bisphosphate redistribution-based sensing mechanism initiates a phagocytosis programing
Source: Nat Commun. 2018 Oct 15;9:4259. doi: 10.1038/s41467-018-06744-7 (PMC6189171; doi:10.1038/s41467-018-06744-7)
Supplement: Supplementary file 3 — Description of Additional Supplementary Files [file 41467_2018_6744_MOESM3_ESM.pdf]

Description of Additional Supplementary Files

File Name: Supplementary Data 1
Description: Corresponding genes identified in the mouse genome from PROSITE (http://prosite.expasy.org/) using Tyr-X-X-(Leu/Ile)-X(6-12)-Tyr-X-X-(Leu/Ile) as the probe.

File Name: Supplementary Data 2
Description: mRNA top expressers in the TABLE 1 genes identified from NCBI GEO RNA-seq databases (https://www.ncbi.nlm.nih.gov/geo/). Their expression levels are ranked by abundance.

File Name: Supplementary Movie 1
Description: A Phagocytic cup enriched with Moesin and actin was reconstructed in 3D with Imaris 7.2.3. Moesin is pseudocolored green with 30% transparency for better viewing and actin is pseudocolored red.

File Name: Supplementary Movie 2
Description: Sub-cellular localization of PH-GFP in response to PIP2 sequestration by 0.1 or 10 mM Geneticin over a 40 mins time interval. Videos are shown at an 8 fps playback speed. Geneticin was added at t=0 mins.

File Name: Supplementary Movie 3
Description: An overall comparison of differences and similarities between PIP2 redistribution and FcR-based phagocytosis mechanisms.
